# Rhamnolipid coating reduces microbial biofilm formation on titanium implants: an in vitro study

**DOI:** 10.1186/s12903-021-01412-7

**Published:** 2021-02-04

**Authors:** Erica Tambone, Emiliana Bonomi, Paolo Ghensi, Devid Maniglio, Chiara Ceresa, Francesca Agostinacchio, Patrizio Caciagli, Giandomenico Nollo, Federico Piccoli, Iole Caola, Letizia Fracchia, Francesco Tessarolo

**Affiliations:** 1 Department of Industrial Engineering and BIOtech, University of Trento, via Sommarive, 38123 Trento, Italy; 2 Department of Laboratory Medicine, Azienda Provinciale Per I Servizi Sanitari, 38122 Trento, Italy; 3 Department CIBIO, University of Trento, 38123 Trento, Italy; 4 Department of Pharmaceutical Sciences, Università del Piemonte Orientale “A. Avogadro”, 28100 Novara, Italy; 5 Healthcare Research and Innovation Program (IRCS-FBK-PAT), Bruno Kessler Foundation, 38123 Trento, Italy

**Keywords:** Dental implants, Biofilm, Titanium, Biosurfactant, *Staphylococcus* spp

## Abstract

**Background:**

Peri-implant mucositis and peri-implantitis are biofilm-related diseases causing major concern in oral implantology, requiring complex anti-infective procedures or implant removal. Microbial biosurfactants emerged as new anti-biofilm agents for coating implantable devices preserving biocompatibility. This study aimed to assess the efficacy of rhamnolipid biosurfactant R89 (R89BS) to reduce *Staphylococcus aureus* and *Staphylococcus epidermidis* biofilm formation on titanium.

**Methods:**

R89BS was physically adsorbed on titanium discs (TDs). Cytotoxicity of coated TDs was evaluated on normal lung fibroblasts (MRC5) using a lactate dehydrogenase assay. The ability of coated TDs to inhibit biofilm formation was evaluated by quantifying biofilm biomass and cell metabolic activity, at different time-points, with respect to uncoated controls. A qualitative analysis of sessile bacteria was also performed by scanning electron microscopy.

**Results:**

R89BS-coated discs showed no cytotoxic effects. TDs coated with 4 mg/mL R89BS inhibited the biofilm biomass of *S. aureus* by 99%, 47% and 7% and of *S. epidermidis* by 54%, 29%, and 10% at 24, 48 and 72 h respectively. A significant reduction of the biofilm metabolic activity was also documented. The same coating applied on three commercial implant surfaces resulted in a biomass inhibition higher than 90% for *S. aureus*, and up to 78% for *S. epidermidis* at 24 h.

**Conclusions:**

R89BS-coating was effective in reducing *Staphylococcus* biofilm formation at the titanium implant surface. The anti-biofilm action can be obtained on several different commercially available implant surfaces, independently of their surface morphology.

## Background

Since the late 70s, the use of osseointegrated implants for replacing missing teeth has become a clinical practice increasingly common and accessible to the population. Dental implants are used in the treatment of partially or totally edentulous patients, with a high success rate [1–4]. Over the years, innovations in oral implantology have concerned both the surgical technique and the implantable materials to make dental implant therapy more and more predictable and effective [5]. Up to date, millions of implants are placed every year worldwide and their survival rate is above > 90% over a 10-year period [3, 6]. However, oral implantology is not free from complications [7–9]. The past three decades have seen the emergence of two relevant oral diseases: peri-implant mucositis and peri-implantitis [10–13]. These peri-implant inflammatory diseases are induced and sustained by the microbial biofilm formed at the surface of the implant components and represent serious complications affecting both hard and soft surrounding tissues [13].

The prevalence of peri-implant diseases represents a debated issue since data deriving from different scientific studies are divergent and indicate a wide range in prevalence, making it difficult to globally estimate the magnitude of the problem [12]. In a systematic review with meta-analyses, Derks & Tomasi tried to clarify this issue and reported a mean prevalence (range) of 43% (19–65%) for peri-implant mucositis and of 22% (1–47%) for peri-implantitis [14]. These data indicate that approximately a fifth of all inserted dental implants are suffering from the peri-implantitis disease, which may lead, over time, to implant loosening or to the need for implant removal [13–15].

Given the difficulty to regain implant osseointegration after peri-implantitis, in the last few years, materials and strategies have been developed and tested to prevent the disease onset by inhibiting or reducing bacterial colonization and promoting at the same time the close contact and growth of peri-implant body tissues with the implant surface. Such strategies can act through different mechanisms including anti-adhesive treatments able to modify implant surface energy and bacterial adhesion, surface coatings with molecules having biocidal or photocatalytic activity, or capable to sustain a controlled release of antimicrobial and/or antiseptic agents at the implant/tissue interface [16–18]. Despite the antimicrobial effectiveness of some implant coatings, there are relevant limitations such as a limited efficacy over time, the possible induction of antibiotic and/or antiseptic resistance, a decreased biocompatibility or even the cytotoxicity of some antimicrobial agents [19–22].

Microbial biosurfactants (BS) represent an interesting class of natural compounds with anti-biofilm and antimicrobial properties. These molecules can be applied to coat implants surface, preserving biocompatibility thanks to their low toxicity [23, 24]. The amphiphilic structure of these molecules can alter the interaction between microorganisms and surfaces, interfering with the microbial adhesion process, and is also able to modify the permeability of the cell membranes, causing a loss of nutrients that leads to cell lysis [24, 25].

In recent years, among naturally derived biosurfactants, rhamnolipids, mainly produced by *Pseudomonas aeruginosa*, have attracted a lot of interest thanks to their biological properties applicable in the biomedical field [26–28].

## Methods

### Aim of the study

This study aimed at developing a new non-cytotoxic coating on medical-grade titanium based on rhamnolipid biosurfactant and testing its efficacy in reducing the amount of microbial biofilm formation. In vitro quantitative tests with two standard biofilm former staphylococcal strains were used to identify the best coating process and generate quantitative data on biofilm inhibition along time. Additional tests were realized on different commercial titanium surfaces in order to provide a proof of principle for the application of the proposed coating to a finished dental implant component.

### Study design

To address this study aim, we structured the experimental design into three phases: (1) optimization of the BS-coating process, (2) investigation of the anti-biofilm efficacy of BS-coated titanium discs at different time-points, (3) testing the anti-biofilm efficacy of the BS-coating deposited on commercial surface morphologies used for dental implants.

Phase 1 was performed by running controlled experiments comparing the biofilm growth at 24 h on laboratory polished titanium surfaces coated using BS solutions at different concentrations ranging from 0 mg/mL (uncoated controls) to 4 mg/mL. Two representative biofilm former *Staphylococcus* spp. strains were considered in the study. Experiments were performed in quadruplicate using the crystal violet (CV) method to quantify the total biofilm biomass formed on coated discs compared to uncoated controls. In addition, a cytotoxicity test was performed on titanium discs coated with the highest BS concentration (4 mg/mL) to obtain preliminary data on their biocompatibility.

Phase 2 addressed the anti-biofilm efficacy of laboratory polished and BS-coated discs with the optimal process at longer time-points. The CV method and the 3-(4,5-dimethylthiazolyl-2-yl)-2,5-diphenyltetrazolium bromide reduction assay (MTT) were used to quantify both the total biofilm biomass and the metabolic activity of the biofilm formed after 24, 48 and 72 h of incubation. CV tests were repeated in three experimental sets, having four replicates for each set. MTT tests, were performed in quadruplicate on a single set. Data were analyzed in order to provide the percentage of biofilm inhibition at the different time-points for each of the two experimental strains.

Phase 3 implemented the optimal coating process on three commercial titanium surface morphologies, representative for a variety of dental implants available on the market. Anti-biofilm efficacy of coated commercial surfaces was assessed by quantifying the biofilm biomass by CV method at 24 h and calculating the percentage of biofilm inhibition with respect to equivalent uncoated controls. Tests were carried out in triplicate on a single experimental session.

Details on the BS production, biofilm former strains selection, biofilm formation, and tests for quantifying biofilm amount and coating anti-biofilm efficacy are reported below.

### Biosurfactant production

The biosurfactant R89 (R89BS) was obtained from the rhamnolipids-producing strain *Pseudomonas aeruginosa* 89 [29]. R89BS was produced, extracted and chemically characterized as described by Ceresa et al. [29]. Briefly, a loop of *P. aeruginosa* 89 overnight culture was inoculated into 40 mL of Nutrient Broth II (Sifin Diagnostics GmbH, Berlin, Germany) and incubated at 37 °C for 4 h at 140 rpm. Afterwards, 24 mL of the seed culture were inoculated in 1.2 L of Siegmund–Wagner medium and incubated at 37 °C for 5 days at 120 rpm. The bacterial cells were removed by centrifugation (Sorvall RC-5B Plus Superspeed Centrifuge, Fisher Scientific Italia, Milano, Italy) at 7000 rpm for 20 min and the supernatant was acidified with 6 M H_2_SO_4_ at pH 2.2, stored overnight at 4 °C and extracted three times with ethyl acetate (Merck KGaA, Darmstadt, Germany). The organic phase was anhydrified and evaporated to dryness under vacuum conditions and the composition of the raw extract confirmed by mass spectrometry analysis as reported previously [29].

### Medical-grade titanium discs and surface characteristics

Titanium alloy Ti6Al4V (medical-grade 5) discs (TDs) with four different surface finishing processes and morphologies were considered in this study. TDs 10 mm in diameter and 2 mm in thickness were obtained from computer numerical control machining and were subsequently polished in the laboratory with increasing fine-grained silicon-carbide abrasive paper up to 4000 grit to obtain a flat surface. Laboratory polished discs were used for optimizing anti-biofilm coating (Phase 1) and evaluating the microbial inhibition efficacy along time (Phase 2). To remove impurities and grinding residues, laboratory polished TDs were cleaned by sonication for 15 min each in three consecutive solutions, 100% acetone, 70% v/v ethanol in distilled water and 100% distilled water, as indicated in Ghensi et al. [30]. The discs were then disinfected by immersion for a minimum of 24 h in 70% v/v ethanol in water and stored in these conditions until further use. TDs were dried under a laminar flow immediately before testing.

In addition to laboratory polished TDs, three commercial micro-morphologies were considered in this study to test efficacy of the R89BS-coating on representative commercial titanium surfaces used in real manufacturing processes for dental implants. For these tests, discs 10 mm in diameter and 1 mm in thickness were provided as sterile coupons from dental implants manufacturer companies with the following surface morphologies on a single face of the disc: computer numerical control machined and polished (M&P) flat surface for transmucosal implant components (CLC Scientific, Vicenza Italy), Laser-Lok® (L-L) micro-treaded medium-roughness surface for trans-mucosal implant components (BioHorizons, Birmingham AL, USA) and Resorbable-Blast Texturing (RBT) blasted high-roughness surface for bone contacting dental components (BioHorizons, Birmingham AL, USA). Discs with commercial surfaces were stored at environmental temperature within the original packaging until experimental use.

### Surface coating process

Coating of TDs surface was performed in 24-well polystyrene plates, fitting one disc for each well. A solution of R89BS in sterile phosphate buffer saline (PBS) was freshly prepared before use according to the desired concentration of BS, ranging from 2 to 4 mg/mL. One milliliter of the desired R89BS solution was added to each well and R89BS-coating was obtained by physical adsorption of the biosurfactant at the titanium surface for 24 h at 37 °C. During coating process, the polystyrene plates containing the samples were agitated at 70 rpm on an orbital shaker. Uncoated control discs having the same surface morphology did not undergo any coating treatment.

Phase 2 and Phase 3 experiments were performed respectively on laboratory polished and commercial titanium discs coated using a 4 mg/mL R89BS solution in sterile PBS at 37 °C for 24 h at 70 rpm. Untreated discs having the same surface morphology were used as controls.

At the end of the immersion period, test discs were aseptically transferred to new 24-well polystyrene plates and dried under a laminar flow to set the BS-coating at the surface.

### Cytotoxicity of R89BS-coated titanium

The potential cytotoxicity of R89BS-coated TDs was evaluated using a previously reported method [29]. Briefly, a lactate dehydrogenase (LDH) assay (ISO 10993) (TOX7 In Vitro Toxicology Assay Kit, Sigma-Aldrich, Darmstadt, Germany) was performed using normal lung fibroblasts (MRC5), according to TOX7 operative procedures. The cell line was maintained in modified Eagle’s medium (MEM) supplemented with 10% fetal calf serum (FCS), l-glutamine (2 mM), sodium pyruvate (1 mM), 1% non-essential amino acids, and 1% antibiotics at 37 °C, 5% CO_2_, and 95% relative humidity.

Titanium discs coated with 4 mg/mL R89BS were immerged in fresh cell culture medium at 37 °C for 24 h in dynamic conditions, obtained by orbital shaking at 1 Hz, to favor biosurfactant removal from the surface. At the same time, cells were seeded in 96-well tissue culture plates and cultured in standard medium until about 70% confluence (24 h). The growth medium was then removed and replaced with the conditioned surface-contacting medium (200 µL/well).

The cytotoxic effect was measured on the basis of the amount of LDH released by cells after 48 h of exposure to the surface contacting medium. The positive control for cytotoxicity was constituted by fully lysate cells after exposure to 0.5% Triton X. Negative control was obtained from cells in reduced medium without surfactant. LDH level was evaluated by light absorbance at 490 nm (Tecan Spark 10 M). Assays were carried out in quintuplicate per each test condition.

### Biofilm growth on titanium discs

Two reference strains, *Staphylococcus aureus* ATCC 6538 and *Staphylococcus epidermidis* ATCC 35984, were used in this study, given their ability of producing high amount of slime according to the methods and criteria proposed by Christensen et al. [31] and subsequently detailed by Stepanovic and co-workers [32]. Strains were stored at − 80 °C in Tryptic Soy Broth (TSB) (Scharlab Italia, Milano, Italy) supplemented with 25% glycerol and grown on Tryptic Soy Agar (TSA) plates at 37 °C for 20 h before experimental assay.

*Staphylococcus aureus* ATCC 6538 and *S. epidermidis* ATCC 35984 suspensions at the concentration of 1 × 10^7^ Colony Forming Unit per mL (CFU/mL) were prepared in TSB supplemented with 1% w/v glucose (Scharlab Italia) to induce slime production [33].

Biofilm formation on the surface of the titanium discs (both coated and uncoated control discs) was obtained in 24-well polystyrene plates fitting one disc for each well. One milliliter of bacterial suspension was added to each well, thus guaranteeing submersion of the disc into the medium with bacteria. Plates were then immediately incubated at 37 °C for 24 h at 70 rpm in air.

In case the planned incubation period lasted 48 or 72 h, discs were aseptically transferred every 24 h into a new plate containing 1 mL of sterile TSB supplemented with 1% w/v glucose to provide fresh nutrients for the sessile bacterial cells. At the end of the incubation period, the suspension was removed using a micro-pipette, and the discs were gently washed twice with sterile PBS to remove non-adherent cells.

### Quantitative tests for biofilm formation

CV test was used in this study to measure biofilm biomass formed on coated or uncoated samples at the desired time-points. All study phases made use of CV test as a first-line quantitative assay. CV test was performed after drying the biofilm at the discs surface in a laminar flow cabinet. Each disc was dipped in 1 mL of 0.2% w/v crystal violet (CV) solution for 10 min. An additional set of coated discs that did not underwent incubation for biofilm formation was used as blank in each testing session. After removing the CV solution, the discs were washed with distilled water to remove dye excess and air-dried again. The CV bound to the biofilms was then released from the matrix by adding 1 mL of 33% v/v acetic acid (Scharlab Italia) in water.

In Phase 2 a second quantitative test, the MTT reduction assay, was implemented to obtain complementary information about the sessile bacteria organized at the titanium surface. This test was realized by immersing each disc and the biofilm in the hydrated state in 1 mL of a 0.075% w/v MTT solution (Fisher Scientific Italia, Milano, Italy). Five microliters of glucose solution (20% w/v in distilled water) and 10 µL of 1 mM menadione solution (Sigma-Aldrich, Milan, Italy) were then added, and samples were incubated for 30 min at 37 °C. Coated discs without biofilm were also included in each MTT session as blanks. Finally, the formazan crystals were dissolved in 1 mL of a lysing solution composed by 7 parts of dimethyl sulfoxide (Scharlab Italia) and 1 part of 0.1 M glycine buffer (pH 10.2) (Sigma-Aldrich).

CV and MTT resulting solutions were spectrophotometrically read at 570 nm (Victor^3^V™, Perkin Elmer, Milano, Italy).

### Scanning electron microscopy of titanium surfaces and bacterial biofilms

A qualitative micro-morphological analysis of the three commercial titanium surfaces investigated in Phase 3, and of the *Staphylococcus* spp. biofilm formed at 24 h with and without the R89BS-coating was carried out by scanning electron microscopy (SEM), as described in Ceresa et al. [34] with minor modifications. Original untreated commercial surfaces were directly mounted on aluminum stubs using double-sided carbon-conducting tape and imaged without further preparation. Discs with biofilm were dipped in 1 mL of 2.5% w/v glutaraldehyde solution in 0.1 M phosphate buffer for 24 h at 4 °C to preserve the microstructural architecture of the biofilm on the titanium surface. Then, each disc was washed twice with Milli-Q® water, dehydrated by immersion in 70%, 90% and 100% v/v ethanol/water solutions for 10 min each and finally dried overnight under a laminar flow cabinet. Dried samples were then coated by a 10-nm layer of gold using a sputter coater (Emitech K500X, Quorum Technologies, Laughton, UK) to improve their electrical conductivity and thermal stability.

SEM observation was performed using a XL30 (FEI-Philips, Eindhoven, The Netherlands) scanning electron microscope in the high-vacuum mode. A set of four images for each disc were obtained by collecting the secondary electron signal at a magnification of 500 × , 1000 × , 2000 × , and 4000 × in order to detect both the titanium surface morphology and the fine structural detail of the microbial cells and of the extracellular matrix on the biofilm. The primary beam energy was set to 10 kV for the titanium surfaces and was lowered to 5 keV when biofilm was present to minimize damage to the organic structures. Possible artefacts due to the sample preparation process [35] were considered according to indications provided by Hrubanova et al. [36] and previous experience performed in imaging microbial biofilm formed in vitro on medical devices [37–39] and in vivo on titanium abutments [40, 41].

### Data analysis and statistics

The single titanium disk was considered as statistical unit. Considering that biological data from CV assays or MTT assays are usually not normally distributed, quantitative data obtained from replicated CV and MTT were expressed as medians and interquartile ranges.

Mann–Whitney U test followed by Bonferroni *post-hoc* test were used to evaluate the effect of the different concentrations of R89BS on *Staphylococcus* spp. biofilm formation and to study the significance of data in the LDH cytotoxicity assay in comparison to positive and negative controls.

The percentage of cytotoxicity was calculated as follows:

1 $$Cytotoxicity \left(\%\right)=\left(\frac{{A}_{R89BS}}{{A}_{pos.Ctrl}}\right)\times 100$$

where A_R89BS_ is the absorbance value of samples treated with R89BS and A_pos.Ctrl_ is the absorbance value of positive control (0.5% Triton X).

The effect of R89BS-coated TDs on *Staphylococcus* spp. biofilm formation was investigated with Mann–Whitney U test, comparing coated discs with uncoated controls at each time-point.

Results were considered to be statistically significant when *p* < 0.05. Statistical analysis was elaborated by means of the statistical program R, 3.5.3 (R Development Core Team, http://www.R-project.org).

Further, CV and MTT data were normalized with respect to the value of the corresponding blank and the inhibition percentages of biofilm formation was determined using the following formula:

2 $$Inhibition \left(\%\right)=\left(1-\left(\frac{{A}_{R89BS}}{{A}_{Ctrl}}\right)\right) \times 100$$

where A_R89BS_ is the absorbance value of BS-coated samples and A_Ctrl_ is the absorbance value of the untreated control. Inhibition percentages for biofilm biomass and metabolic activity of sessile microbial cells were provided in tabular form for ease of comparison between different time-points and different commercial surfaces.

## Results

### Optimized process for coating titanium with R89 biosurfactant

The biofilm biomass formed on TDs coated using solutions at different concentrations of R89BS (0, 2 and 4 mg/mL) was obtained for *S. aureus* ATCC 6538 and *S. epidermidis* ATCC 35984 at 24 h.

**Fig. 1:**
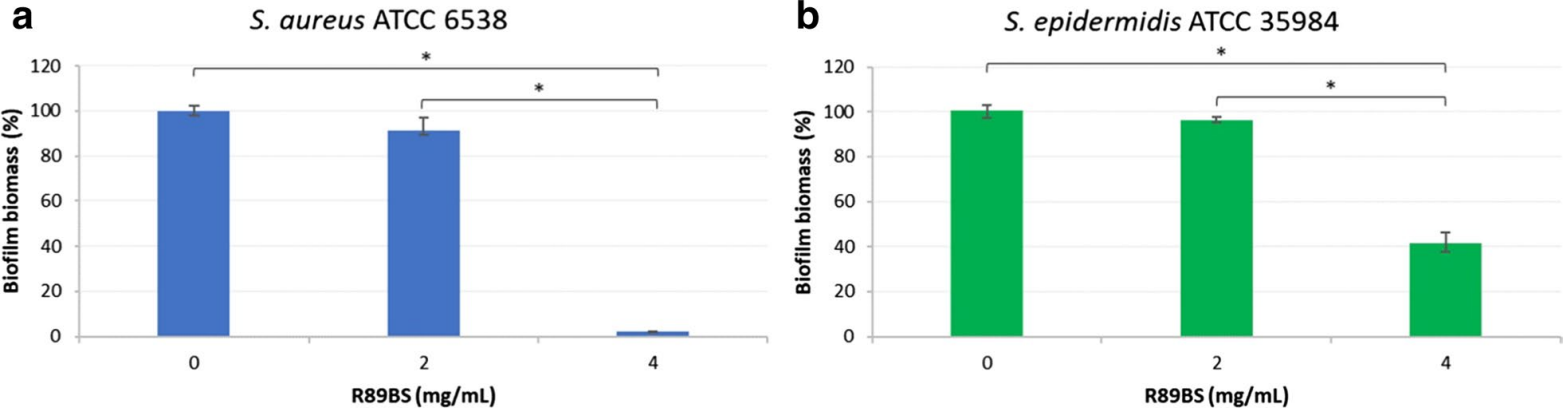
*S. aureus* (**a**) and *S. epidermidis* (**b**) biofilm formation on laboratory polished titanium discs coated by physical adsorption with different concentrations of R89BS (0, 2 and 4 mg/mL). Data are obtained from CV staining after 24 h incubation. Results are displayed with respect to uncoated controls (0 mg/mL), normalized to 100% for ease of comparison. Error bars represent interquartile ranges around median values. *p < 0.05

For both *Staphylococcus* strains, the use of a 2 mg/mL R89BS solution in the coating process did not result in a significant reduction of the biofilm amount with respect to uncoated controls (0 mg/mL). On the contrary, a significantly lower amount of biofilm biomass was observed by increasing the concentration of R89BS to 4 mg/mL (Fig. 1). A median (first quartile, third quartile) biofilm inhibition of 98.9 (97.7; 98.3)% for *S. aureus* and 56.8 (54.0; 60.3)% for *S. epidermidis* was obtained using the concentration of 4 mg/mL (*p* < 0.05). Based on these results, the coating of the titanium surface using a solution of 4 mg/mL R89BS was considered as the optimal process, requiring the minimal amount of R89BS to produce a significant anti-biofilm effect with both the reference biofilm producer strains.

### Cytotoxicity of R89BS-coated TDs

**Fig. 2:**
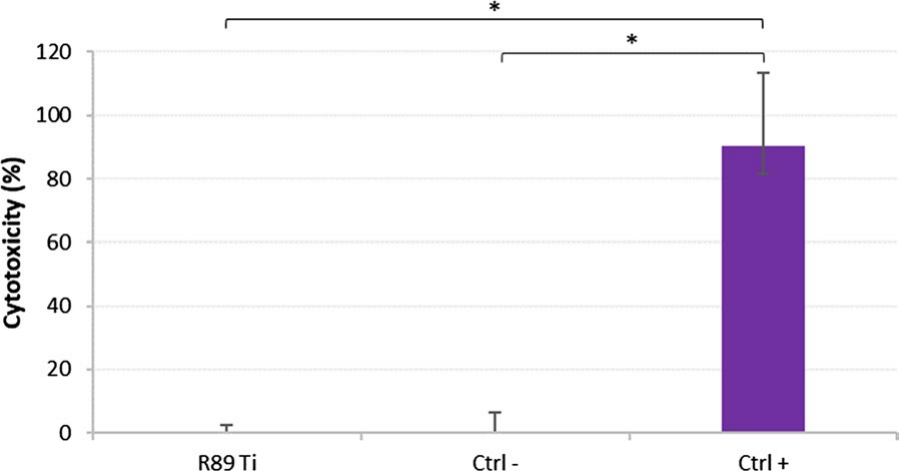
Cytotoxicity of R89BS-coated TDs on human lung fibroblast (MRC5). Positive control (Ctrl+) is represented by fully lysate cells (0.5% Triton X) and negative control (Ctrl−) is represented by cells in standard growth medium. No significant difference in cytotoxicity was found between negative control (Ctrl−) and data obtained using the growth medium used for eluting the BS from the titanium discs surface (R89Ti). Error bars represent interquartile ranges around median values. *p < 0.05

The cytotoxicity assay showed no cytotoxic effect on human lung fibroblasts cell lines when exposed to the TDs coated with 4 mg/mL R89BS eluate obtained from dynamic release conditions. After 48 h of co-incubation, MRC5 cells viability was comparable to negative controls (growth medium), as illustrated in Fig. 2.

### Anti-biofilm activity of R89BS-coating

The ability of laboratory polished TDs coated with the optimal coating process (4 mg/mL R89BS) to inhibit *Staphylococcus* spp. biofilm formation was further investigated by CV and MTT tests performed at 24, 48 and 72 h of incubation.

**Fig. 3:**
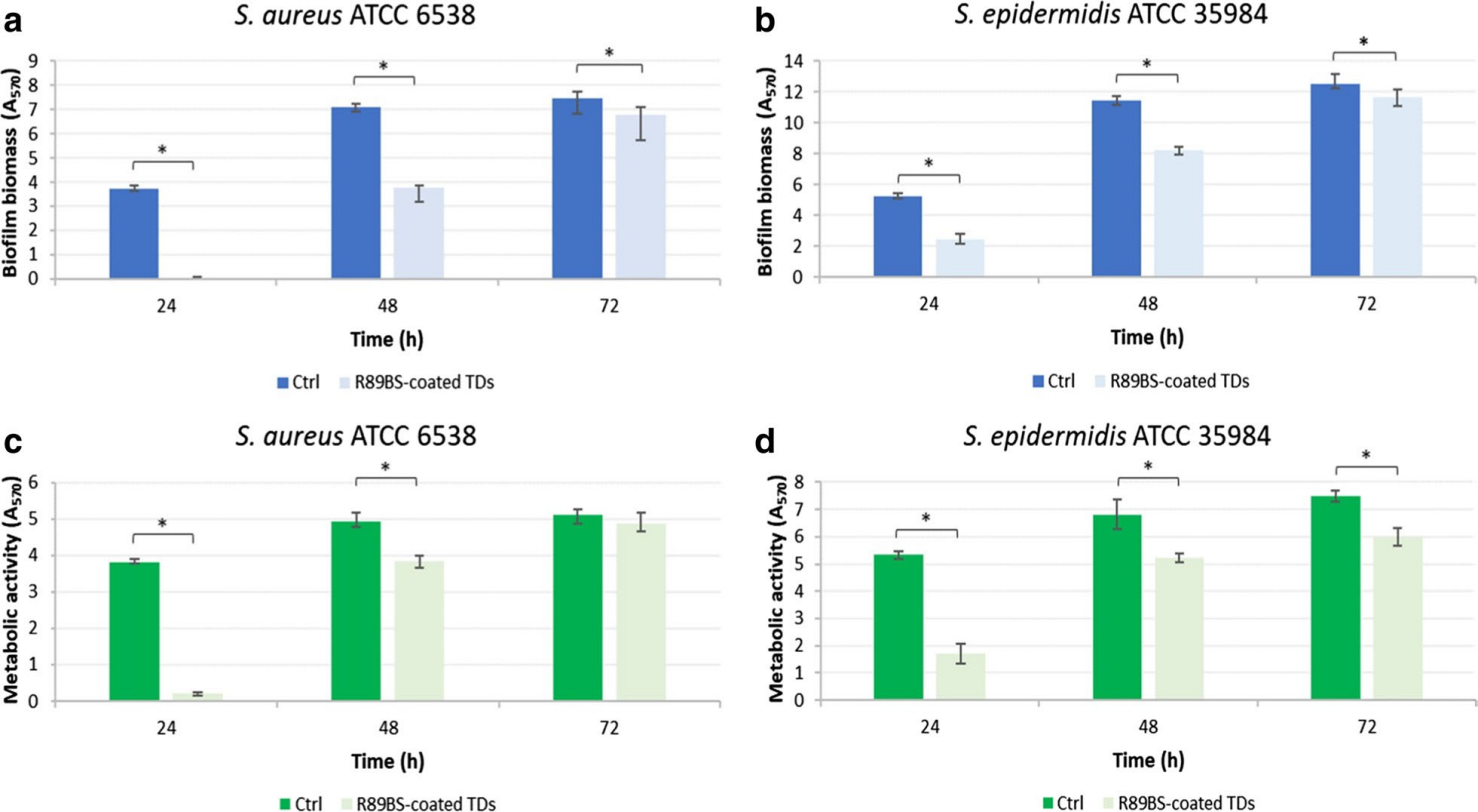
*Staphylococcus* spp. biofilm formation on polished titanium discs coated with 4 mg/mL R89BS (light bars) with respect to uncoated controls (dark bars). Results are presented both in terms of biofilm biomass (**a**, **b**), determined by CV assay, and cell metabolic activity (**c**, **d**), determined by MTT assay, after 24, 48 and 72 h of incubation. Error bars represent interquartile ranges around median values. *p < 0.05

From a qualitative point of view, the inspection of data reported in Fig. 3 shows that both biofilm biomass and biofilm metabolic activity increased on time for both control and coated discs. However, a significant difference in the amount of biofilm biomass is present for both tested strains at all the tested time-points, showing a marked less amount of biofilm on coated surfaces. A similar trend was obtained from the MTT test data, showing a lower metabolic activity in biofilms grown on coated titanium surfaces in all experimental conditions. Statistical significance was obtained in all but one experimental condition (*S. aureus* biofilm after 72 h of incubation). Differences were also present when comparing the biofilm formation of the two different test strains. For *S. aureus*, R89BS-coating resulted more effective in reducing biofilm biomass (Fig. 3a, c). On the contrary, *S. epidermidis* biofilm was mostly inhibited in terms of cell metabolic activity (Fig. 3b, d).

From a quantitative perspective, R89BS-coated TDs showed the highest ability to reduce biofilm formation at 24 h of both *Staphylococcus* strains. At this time-point, the calculated inhibition of the biofilm biomass and cell metabolic activity reached 98.6% and 94.3% for *S. aureus,* and 54.1% and 68.9% for *S. epidermidis*, respectively (*p* < 0.001). The anti-biofilm activity of coated TDs gradually decreased over time, resulting in an overall biomass inhibition of 7.0% for *S. aureus* and 10.3% for *S. epidermidis*, at 72 h. Table 1 reports the percentages (medians and interquartile ranges) of biomass and metabolic activity inhibition at the different time-points.

**Table 1:** Biofilm biomass and cell metabolic activity inhibition percentages of *Staphylococcus* spp. determined by R89BS-coated TDs at 24, 48 and 72 h

Strain	Incubation time (h)	Biomass inhibition (%) Median (first quartile; third quartile)	Cell metabolic activity inhibition (%) Median (first quartile; third quartile)
*S. aureus*	24	98.6 (98.2; 99.2)	94.3 (93.8; 95.5)
	48	46.9 (45.5; 50.2)	25.7 (18.5; 31.4)
	72	7.0 (3.4; 17.7)	0.2 (− 3.9; 3.9)
*S. epidermidis*	24	54.1 (49.7; 57.0)	68.9 (66.7; 70.7)
	48	29.3 (26.6; 31.0)	23.6 (20.4; 28.8)
	72	10.3 (5.8; 12.4)	19.1 (16.3; 22.8)

### Efficacy of R89BS-coating on commercial titanium surfaces

The ability of R89BS-coating to inhibit *Staphylococcus* spp. biofilm formation was finally assessed on three commercially available titanium surfaces (M&P, L-L, and RBT) used for realizing dental implants. The reduction of biofilm biomass on R89BS-coated TDs with respect to control discs was evaluated by CV staining after 24 h.

**Fig. 4:**
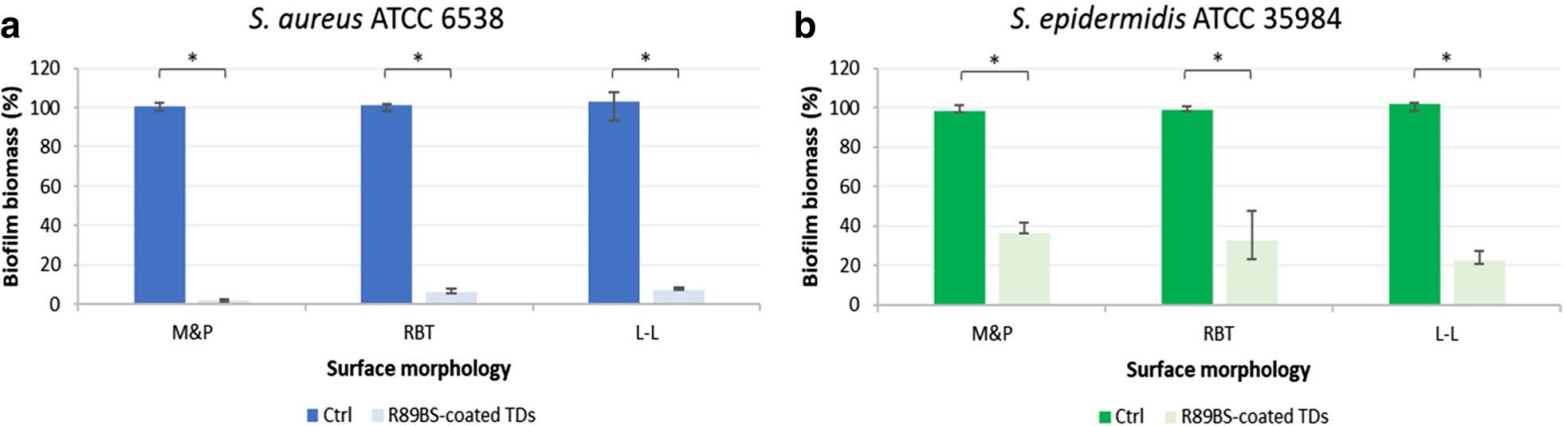
*S. aureus* (**a**) and *S. epidermidis* (**b**) biofilm biomass formed on three different commercial titanium surfaces coated with 4 mg/mL R89BS. Data are obtained from CV staining after 24 h incubation. Results are displayed with respect to uncoated controls, normalized to 100% for ease of comparison. M&P, computer numerical control machined and polished; RBT, resorbable-blast texturing blasted; L-L, Laser-Lok® micro-treaded. Error bars represent interquartile ranges around median values. *p < 0.05

The three different titanium surfaces coated with R89BS showed a comparable anti-biofilm effect (Fig. 4). *S. aureus* biofilm biomass was inhibited by more than 90% (*p* < 0.001) in R89BS-coated samples with respect to uncoated controls, irrespective of the surface morphology (Fig. 4a). For *S. epidermidis*, a significant biofilm inhibition was obtained for all coated surfaces with respect to uncoated controls with an inhibition percentage ranging from 62 to 78%, according to the specific surface morphology (Fig. 4b). The percentages (medians and interquartile ranges) of inhibition obtained by coating with R89BS the three commercial morphologies are summarized in Table 2.

**Table 2:** Inhibition percentages of *Staphylococcus* spp. biofilm biomass determined by coating with R89BS titanium discs with three different commercial surface morphologies: computer numerical control machined and polished (M&P), resorbable-blast texturing blasted (RBT), and laser-Lok® micro-treaded (L-L)

Surface morphology	Biomass inhibition (%) Median (first quartile; third quartile)	
	*S. aureus*	*S. epidermidis*
M&P	97.8 (97.6; 98.4)	62.4 (57.2; 63.8)
RBT	93.5 (91.8; 94.2)	66.5 (51.7; 76.6)
L-L	91.3 (91.2; 92.2)	78.1 (73.5; 78.8)

**Fig. 5:**
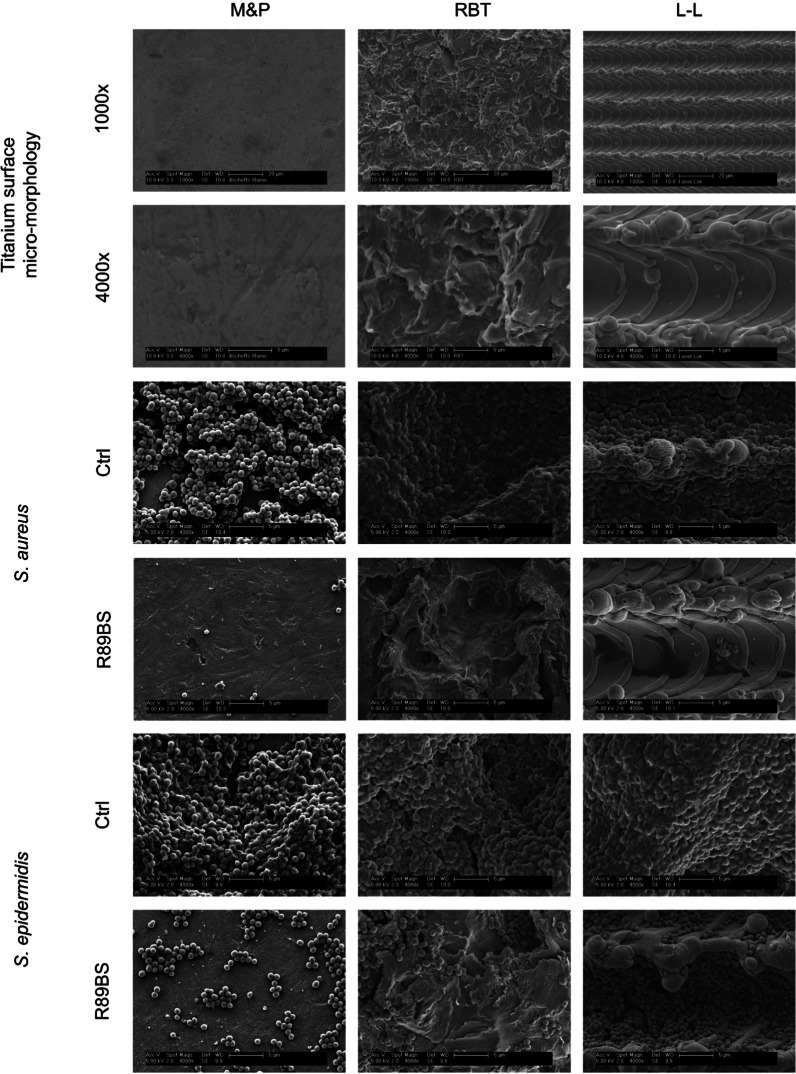
Topographical details of the three commercial titanium morphologies considered in this study (first and second rows) and biofilm architectures of *Staphylococcus* spp. cells adhered at 24 h on the same surfaces coated with 4 mg/mL R89BS (R89BS) or uncoated (Ctrl). M&P: computer numerical control machined and polished; RBT, resorbable-blast texturing blasted; L-L, laser-Lok® micro-treaded. Representative images were obtained by scanning electron microscopy in high-vacuum mode. Original magnification: × 1000 (first row), × 4000(other rows)

The SEM analysis evidenced the peculiar topographic characteristics of the three commercial surfaces. The M&P finishing process resulted in a flat surface, showing minor groves and indentations with sub-micrometric depth, arranged in a random orientation due to the final polishing. On the contrary, RBT surface was characterized by a high roughness, formed by a continuum of crevices and craters in the range from 1 to 20 µm, with irregularly shaped and sharp boundaries resulting from the blasting process. L-L was characterized by an anisotropic finishing pattern, with an overall intermediate roughness. The pulsed-laser process induced the creation of parallel regular grooves, spaced about 20 µm, to form a micro-treaded oriented surface characterized by minor irregularities with smoothed edges. A representative field of view at 1000 × and 4000 × for each surface is reported in the first and second row of Fig. 5 respectively. In the same figure, we report a selection of representative fields of view for the biofilm formed at the surface of the uncoated (controls) and R98BS-coated discs after 24 h of incubation. The difference in the number of sessile bacteria and in the biofilm volume between coated and uncoated surface is obvious and in agreement with quantitative data obtained from CV staining. In the control samples, microbial cells were arranged in mono- and multi-layered structures, covering the large majority of the surface, irrespective of the titanium micromorphology (third and fifth rows of Fig. 5). In coated samples, a markedly lower number of cells was present, either isolated or forming small aggregates at the surface, concentrated preferentially in the titanium crevices and recesses (fourth and sixth rows of Fig. 5). The lowest number of microbial cells at the surface was observed on the R89BS-coated surface incubated with the *S. aureus* strain (fourth row in Fig. 5).

## Discussion

Titanium and its medical-grade alloys, in particular the Ti6Al4V alloy used in this study, represent the materials of choice for realizing a wide range of dental implant components. Due to its excellent biocompatibility and resistance to corrosion, high strength and good mechanical properties [42–44] Ti6Al4V is largely used in dental implantology, especially for transmucosal components where limiting biofilm formation is a crucial factor. Adhesion and biofilm formation at the implant surface due to oral microbiota or pathogenic microorganisms introduced during or after surgery is one of the main causes of dental implants failure [45, 46]. The formation and preservation of an effective mucosal seal around the transmucosal components [47–49] and a quick osseointegration process [49, 50] are essential for limiting the microbial migration to the coronal portion of the implant. Therefore, effective solutions for oral implantology should inhibit microbial cell attachment and biofilm formation at the surface of the implant and of transmucosal components preserving biocompatibility and bioactive properties required for the integration with the peri-implant tissues.

Within the scientific community there is still an open debate about how to address and resolve the problem of peri-implant diseases: some researchers advocate a return to smooth or less rough surfaces [51, 52]. Other authors suggest improving the mucosal seal around implants, being considered the true entrance door for the microorganisms [53, 54]. In addition to this, some scientists believe that a revision of the indications and criteria of modern implantology is needed [55]. Most probably, a multilateral prevention and a structured therapeutic intervention could represent a promising approach. Specific and regular check-ups of the peri-implant tissues, and the evaluation and mitigation of risk factors (e.g. periodontitis, smoking, systemic diseases) are effective precautions [13, 56–58], but still insufficient to have a resolutive impact on the incidence of the disease.

Peri-implantitis is therefore a well-established disease and urges for new strategies, procedures and devices able to counteract or prevent its occurrence. Recently, it has been suggested that new surfaces or coatings with antibacterial or anti-biofilm properties should be developed and tested [59, 60]. Microbial biosurfactants emerged as new of anti-biofilm agents for coating implantable devices preserving their biocompatibility. However, published studies are mainly focused on the evaluation of antimicrobial activity on planktonic cells [61, 62]. A limited number of studies addressed the potential of rhamnolipid biosurfactants in preventing microbial adhesion and biofilm formation on conditioned surfaces [30, 63–66], and to our knowledge, this is the first time that an anti-biofilm rhamnolipid-coating was applied on medical-grade titanium.

Zezzi do Valle Gomes et al. [67] reported that a polystyrene surface pre-coated with a 1.0% aqueous solution of rhamnolipids produced by *P. aeruginosa* LBI reduced by 58% and 68% the adhesion of *Listeria monocytogenes* and *S. aureus* respectively. The anti-biofilm activity of the rhamnolipid biosurfactant produced by *Burkholderia thailandensis* E264 was explored by Elshikh et al. against some typical oral colonizers [68]. An inhibition of biofilm formation of 57% for *Streptococcus oralis*, 70% for *Neisseria mucosa* and *Actinomyces naeslundii* and 83% for *Streptococcus sanguinis* was observed on a polystyrene surface pre-coated with the rhamnolipid at a concentration of 6.25 mg/mL [68].

In a recent work by Ceresa et al. [29], a coating with the biosurfactant R89, the same used in our study, has proven to be effective in inhibiting *Staphylococcus* spp. biofilm formation on medical-grade silicone up to 72 h, with an overall inhibition of 76% for *S. aureus* and 63% for *S. epidermidis*. Mass spectrometry analysis of R89BS revealed that the BS crude extract is a mixture composed by homologues of mono-rhamnolipids (75%) and di-rhamnolipids (25%). In addition to anti-biofilm properties, R89BS showed antibacterial activity on *Staphylococcus* spp. planktonic cells, with a minimal inhibitory concentration (MIC) values of 0.06 mg/mL for *S. aureus* and 0.12 mg/mL for *S. epidermidis* [29]. Stemming from these encouraging results, we addressed the possibility of realizing a coating with R89BS on titanium.

In this study, the rhamnolipids-coating was realized by R89BS physical adsorption at the titanium surface, and its anti-biofilm efficacy was investigated on *Staphylococcus aureus* and *Staphylococcus epidermidis* up to 3 days. *S. aureus* and *S. epidermidis* were selected as they represent the two major bacterial strains responsible of titanium implant-related infections. They are frequently introduced during implants surgery or in the post-operative period, causing infections that generally involve the formation of an antibiotic-resistant biofilm [69].

Different quantitative aspects of microbial biofilm were considered in this study, such as total biomass and cell metabolic activity. The designed experimental conditions allowed the reproducible development of a mature and structured staphylococcal biofilm on the titanium surfaces. The R89BS-coating was able to significantly reduce *Staphylococcus* spp. cells adhesion over time, showing a remarkable effect at 24 h with a biofilm biomass inhibition of more than 98% and 54% for *S. aureus* and *S. epidermidis* respectively. At more prolonged incubation times, the inhibition decreased, but was still able to guarantee a significantly lower amount of biofilm biomass in coated samples compared to uncoated controls at 72 h.

The anti-biofilm activity of R89BS can be related to its ability to reduce titanium hydrophobicity, possibly because of the R89BS molecules orientation at the surface, interfering with the hydrophobic interactions responsible of the initial adhesion of the microbial cells to a solid surface. According to Walencka et al. [70], biosurfactants may affect both the interactions of bacterial cells with each other and with the surface, thanks to their ability to reduce surface tension and to change bacterial cell walls charge. Moreover, at neutral pH condition the carboxylic groups of the fatty acid alkyl chain are mainly in the anionic form, so an electrostatic repulsion is established between the negative charges of the bacterial surface and the negative charges of the biosurfactant molecules on the titanium surface [67, 71].

In this study, the anti-biofilm efficacy of R89BS-coating was also assessed on three representative titanium surfaces used in the manufacturing processes of dental implants and/or implant components (e.g. transmucosal abutments and healing abutment). L-L and RBT disc surfaces, obtained by laser-etching and blasting respectively, are characterized by higher surface roughness compared to the M&P titanium. In literature, it is reported that rougher surfaces support differentiation, growth and attachment of bone cells, and increases mineralization, promoting osseointegration, essential for implants success [72–75]. This is one of the reasons why several different morphologies were developed and deployed on the market and different morphologies are often adopted for different parts of the same implant. However, it seems that an increase in surface roughness also enhances bacterial adhesion and biofilm formation [76–78]. The coating with R89BS resulted effective in inhibiting *Staphylococcus* spp. adhesion on the three tested commercial surfaces, with comparable (for *S. aureus*) or better (for *S. epidermidis*) results with respect to those obtained with laboratory prepared surface, highlighting the potential of this biosurfactant in preventing dental implants colonization, irrespective from their surface morphology.

Another important aspect of R89BS-coated titanium is its low cytotoxicity. Ceresa et al. [29] reported no significant cytotoxicity on eukaryotic cells for R89BS in solution at concentrations less than or equal to 0.2 mg/mL. In this work, further data were provided by using an additional biocompatibility test, closer to the destination of use of the titanium component, by simulating the possible elution of the BS from a titanium device into a liquid medium. No cytotoxic effect was detected when eukaryotic cells were exposed to the eluate from R89BS-coated titanium discs. These data enlarge the body of evidence that support further testing toward in vivo applications.

Despite the interesting results we obtained, there are some limitations of our study that, if addressed, will provide more accurate data. Although *S. aureus* ATCC 6538 and *S. epidermidis* ATCC 35984 are widely recognized as biofilm former strains and positive results with these strains are encouraging, further tests have to be carried out, possibly including biofilm forming strains of relevance for the microbiome of the peri-implant diseases [79, 80]. In that regard, additional investigation could be performed on multi-species biofilms formed on titanium surfaces by commonly initial, early, secondary and late dental colonizers (such as *Streptococcus oralis*, *Veillonella parvula*, *Fusobacterium nucleatum* and *Porphyromonas gingivalis*) in a protein-rich medium in anaerobic conditions as described by Sanchez et al. [81]. Some limitations are however present in realizing robust biofilm model in vitro due to stringent culturing conditions required by the majority of the oral microorganisms. These limitations could be overcome by using a flow chamber system in which biofilms could grow under hydrodynamic conditions and the environment could be carefully controlled and easily modulated [82]. Animal in vivo studies could possibly provide the best testing conditions and should be also considered in future.

A further limitation is represented by the loss of anti-biofilm efficacy in time. Although 72 h could be relevant to protect the implant immediately after surgical placement, a prolonged efficacy is desirable to prevent the onset of the peri-implant diseases at later stages after installation. An explanation of the gradual reduction of R89BS-coating efficacy over time can be found in the nature of the bonds between the titanium surface and the biosurfactant. Physical adsorption is a simple method to coat the titanium for a short time since interactions of the BS molecules with the surface are realized through weak bonds based mainly on hydrophobic and van der Waals interactions. A progressive detachment of R89BS with time is possible when the implant is exposed to an aqueous environment. This gradual loss may generate areas of uneven coating where microbial cells can adhere creating, gradually, thicker biofilms with the consequent observed reduction of activity. Alternative bonding strategies should be assessed in the future to improve durability of R89BS-coating and long-term anti-biofilm efficacy, for example through chemical modification of the titanium surface in order to promote a covalent bonding of the active molecule to the surface.

Eventually, the clinical application of this coating to dental implants, or to titanium transmucosal components, requires several additional tests before being considered safe and effective. Biocompatibility test performed up to now were limited to cytocompatibility assay and cannot guarantee that interaction with bone or soft tissues is not affected by the presence of the R89BS coating. Additional biocompatibility tests with relevant cell lines (e.g.: gingival fibroblasts, osteocytes) have to be considered before animal testing.

A range of possible clinical applications can be envisaged. The R89BS coating can be considered for application not only to the dental implant, but also to other titanium implant components (e.g. titanium transmucosal components). Temporary transmucosal components (e.g. healing abutments) coated by R89BS can also be considered as possible adjuvant in the healing process of an infected gum, having the advantage of being removable and exchanged during the healing process. Moreover, different ways to coat titanium surfaces with R89BS can be considered, ranging from the application of the coating at the end of the component manufacturing process, to the “on site” application realized by the dentist before the implant placement or at implant revision. The surface treatment can be limited to specific areas of the implant component (e.g.: implant shoulder, abutment external surface) by selectively masking other areas before application. This could help preserving peculiar surface characteristics and peri-implant tissue interaction.

## Conclusions

In the present work, a coating with the rhamnolipid biosurfactant R89 was successfully applied on medical-grade titanium samples. Significant anti-biofilm activity was proven up to 72 h in in vitro experiments, representative of worst-case conditions with high concentration of bacterial cells in the inoculum and growing conditions enhancing biofilm formation. Results were shown to be reproducible on both laboratory-prepared surfaces and a range of commercial surface morphologies. Coated titanium samples did not show cytotoxic effect on normal lung fibroblasts (MRC5).

In a summary, R89BS-coating resulted to be a promising strategy to prolong lifetimes of dental implants, limiting staphylococcal adhesion and reducing biofilm formation on titanium surfaces. Stressing that maintenance therapy remains essential from a clinical stand-point, new scenarios for the effective mitigation of the peri-implant diseases are now possible.

## Data Availability

The datasets used and analysed during the current study are available from the corresponding author on reasonable request.

## Abbreviations

BS: Biosurfactants
CV: Crystal violet
MTT: 3-(4,5-Dimethylthiazolyl-2-yl)-2,5-diphenyltetrazolium bromide reduction assay
R89BS: Rhamnolipid biosurfactant R89
TDs: Titanium discs
MRC5: Normal lung fibroblasts cell line MRC5
Rpm: Rotations per minute
Ti6Al4V: Titanium-six-aluminum-four-vanadium
M&P: Machined and polished
L-L: Laser-Lok®
RBT: Resorbable-blast texturing
PBS: Phosphate buffer saline
LDH: Lactate dehydrogenase
MEM: Modified Eagle’s medium
FCS: Fetal calf serum
TSB: Tryptic soy broth
TSA: Tryptic soy agar
SEM: Scanning electron microscopy
Ctrl+: Positive control
Ctrl−: Negative control
